# A Case of Sustained Tumor Regression With MP0274, a Novel DARPin Therapeutic Targeting Human Epidermal Growth Factor Receptor 2 Signaling, in Metastatic Human Epidermal Growth Factor Receptor 2–Positive Breast Cancer After Prior Trastuzumab and Pertuzumab

**DOI:** 10.1200/PO.22.00006

**Published:** 2022-11-03

**Authors:** Stefanie Fischer, Thorsten O. Götze, Aurelius Omlin, Richard D. Baird, Keith M. Dawson, Christof Zitt, Zita Arany, Gaby Tresch, Ulrike Fiedler, Simone Jeger, Samson Fung, Philippe Legenne, Nicolas Leupin, Andreas Schneeweiss, Carlo Fremd

**Affiliations:** ^1^ Kantonsspital St Gallen, Klinik für Medizinische Onkologie und Hämatologie, St Gallen, Switzerland; ^2^ Krankenhaus Nordwest GmbH, Institut für Klinisch-Onkologische Forschung, Frankfurt, Germany; ^3^ Cancer Research UK Cambridge Centre, Cambridge, United Kingdom; ^4^ Molecular Partners AG, Schlieren, Switzerland; ^5^ NBE-Therapeutics AG/Boehringer Ingelheim, Basel, Switzerland; ^6^ Fung Consulting, Eching, Germany; ^7^ National Center for Tumor Diseases (NCT), Heidelberg University Hospital and German Cancer Research Center, Heidelberg, Germany

## Background

The human epidermal growth factor receptor 2 (HER2, ERBB2) is amplified or overexpressed in approximately 20% of breast cancers.^1,2^ *HER2* amplification results in increased homodimerization and HER3 heterodimerization, with HER2/HER3 heterodimers promoting cell proliferation via the mitogen-activated protein kinase/AKT pathway and also evasion of apoptosis via the phosphatidylinositol 3-kinase (PI3K) signaling cascade.^3^ Oncogenic mutations in *PI3KCA* render tumor cells resistant to drugs that block HER2 dimerization, thereby promoting cell survival.^4^ Although several approved HER2-directed agents have shown significant survival improvements for HER2-positive patients,^5-7^ in the metastatic setting, nearly all of these patients will ultimately progress and die of their disease.

**FIG 1.**
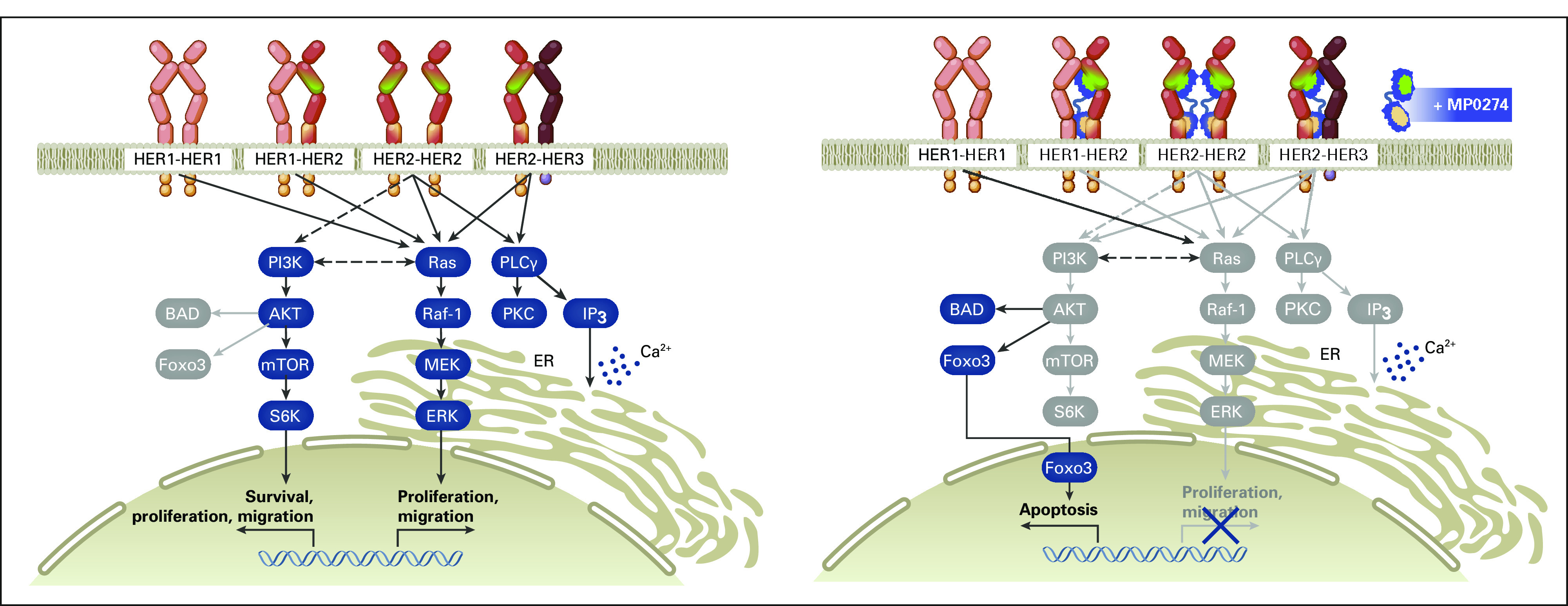
MP0274 mechanism of action inhibiting HER2 signaling. MP0274 binding to HER2 inhibits HER2 signaling of the MAPK/AKT pathway and consequent proliferation, as well as of the PI3K pathway preventing induction of apoptosis. Dark blue and light gray represent phosphorylated and dephosphorylated signaling proteins, respectively. HER2, human epidermal growth factor receptor 2; MAPK, mitogen-activated protein kinase; PI3K, phosphatidylinositol 3-kinase.

Designed ankyrin repeat protein (DARPin) molecules bind targets with high affinity and specificity. Most importantly, they can be combined in a modular fashion to produce multidomain, multifunctional therapeutic proteins, which can be delivered successfully in the clinic.^8^ The trispecific DARPin molecule MP0274 (Baccinex SA, Courroux, Jura, Switzerland) has two domains that bind to different sites on HER2 and two that bind to serum albumin. Its biparatopic binding to HER2 results in a unique proapoptotic mode of action compared with HER2-targeting antibodies, while albumin binding extends its plasma half-life (Fig 1). Binding to HER2 strongly inhibits HER2/HER3 downstream signaling, resulting in inhibition of proliferation and induction of apoptosis in HER2-expressing cancer cell lines.^9^ In breast and gastric HER2-expressing patient-derived xenograft models, MP0274 shows superior antitumor activity compared with trastuzumab and lapatinib, and equivalent efficacy with trastuzumab plus pertuzumab.^10^ MP0274 does not have an Fc domain and so does not induce antibody-dependent cellular cytotoxicity/complement. Preclinical data showed that cell lines harboring activating mutations in the PI3K pathway were resistant to MP0274, suggesting that a wild-type PI3K signaling pathway is necessary for MP0274 activity.

We present here, to our knowledge, the first report of a sustained clinical response in a patient with metastatic breast cancer treated with MP0274. On the basis of preclinical and preliminary biomarker data obtained during a first-in-human dose escalation study evaluating MP0274 in advanced/metastatic *HER2*-positive solid tumors (ClinicalTrials.gov identifier: NCT03084926), we hypothesize that the optimal potential to respond to MP0274 is dependent on a specific genetic signature, requiring both *HER2* amplification and *PI3KCA* wild-type, in addition to adequate MP0274 exposure. Pharmacokinetic (PK) and biomarker data from the dose escalation cohort are presented in the context of this hypothesis. The patient provided written informed consent for publishing the data.

## Case Presentation

A 37-year-old female patient presented in September 2012 with *HER2+* amplified (immunohistochemistry 3+) stage IV (T1N3M1) cancer of the right breast, with liver, bone, and lymph node metastases (axillary, supraclavicular, and hilomediastinal). She was treated with six cycles of docetaxel/pertuzumab/trastuzumab, achieving partial response. Pertuzumab and trastuzumab were continued as maintenance therapy, however, pertuzumab was stopped in April 2013 because of toxicity, and treatment was continued with trastuzumab alone. Disease progression was reported in February 2014 and she started second-line treatment with trastuzumab emtansine, achieving stable disease. In May 2014, she underwent a right mastectomy because of progression of an inflammatory component of the breast tumor. Trastuzumab emtansine was maintained until January 2019, when it was stopped at the patient's request. Trastuzumab emtansine was restarted in January 2020 because of hepatic and lymph node progression; however, further disease progression (mediastinal and axillary lymph nodes) was reported by the end of April 2020 and treatment was stopped.

In June 2020, immunohistochemical and fluorescence in situ hybridization analyses of a fresh tumor biopsy from an axillary lymph node metastasis confirmed *HER2* overexpression and amplification (immunohistochemistry 3+ FISH-amplified per HercepTest and HER2 IQFISH; estrogen receptor 0%, progesterone receptor 0%). Next-generation sequencing was performed (Oncomine Precision Assay, Thermo Fisher Scientific, Waltham, MA) with cell-free tumor DNA (ccfDNA) obtained immediately before treatment, to assess the mutational status of a panel of 50 cancer-related genes, including *HER2*, *HER3*, *HER4*, *TP53*, *PIK3CA*, *PTEN*, *EGFR*, and *AKT1*. This confirmed *HER2* amplification, although no other genetic alterations with potential impact on mode of action (*HER2*, *HER3*, *EGFR* and *PI3K* pathway) were identified, and that she had wild-type *PIK3CA*.

**FIG 2.**
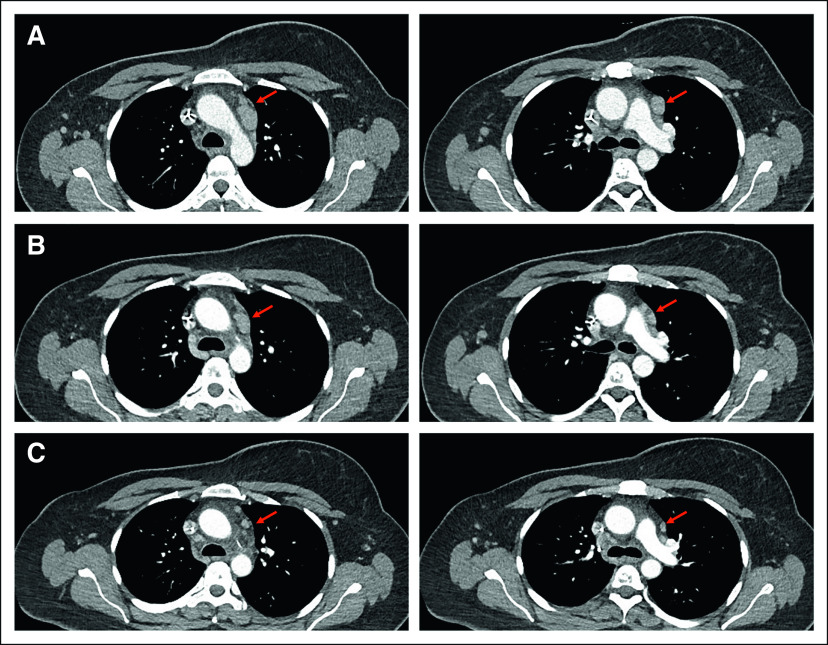
Efficacy of MP0274 in an HER2-positive *PI3K* wild-type breast cancer patient. The patient experienced a sustained partial response in lymph node lesions. Computed tomography scans obtained at (A) baseline and (B) during treatment with 3-week cycles of MP0274 (12 mg/kg) show shrinkage of the lesion at the first scan 2 months later. (C) This tumor reduction was maintained after 14 months of treatment. HER2, human epidermal growth factor receptor 2.

The patient started treatment with 12 mg/kg MP0274 (intravenously, once every 3 weeks, with premedication) in June 2020. A 38% reduction in supraclavicular and hilomediastinal lymph node lesions (measuring 1.7 and 1.5 cm at baseline, respectively) was observed 2 months after the first MP0274 dose, corresponding to a partial response per RECIST v1.1. Response was maintained with continued gradual tumor reduction for both lesions, reaching a maximum 78% shrinkage after 13 cycles (Fig 2). This reduction remained stable for 17 months after initiating MP0274, until progression was reported in December 2021 and trastuzumab deruxtecan was given. The patient maintained a good performance status and quality of life throughout treatment. No signs of cardiotoxicity were observed in repeat echocardiographies. Apart from intermittent mild diarrhea and repeated mild delayed infusion reactions (warmth in cheeks and upper chest), typically occurring approximately 20 hours after infusion, the patient did not experience any adverse effects. *HER2* amplification was confirmed in ccfDNA at cycle 3, but was not detected from cycle 5 onward. No alterations of *PI3K* pathway actors were observed. *TP53* mutations decreased over time, suggesting a reduced tumor mutational burden.

During the dose escalation study, 22 patients were treated over six MP0274 dose levels (0.5-12 mg/kg intravenously, once every 3 weeks; Appendix Table A1). Two events of dose-limiting toxicity, both infusion-related reactions, were reported (0.5 and 8 mg/kg without premedication), and MP0274 was generally well tolerated with minimal treatment delays or reductions. The maximum tolerated dose was not reached. The main toxicity seen across all dose levels was infusion-related reactions (Table 1), with a single grade 3 event. All events were manageable with premedication and standard treatment. Stable disease was reported in three patients, at 0.5, 4, and 8 mg/kg and lasting 8.8, 4.7, and 4.4 months, respectively (Table 2). Retrospective biomarker analyses of ccfDNA revealed that none of these three patients had a combination of *HER2* amplification and *PI3KCA* wild-type (Table 2). This genetic combination was reported in only three patients treated up to 8.0 mg/kg (at 0.5, 1.5, and 8 mg/kg), all of whom had progressive disease as best response. Furthermore, the patient treated at 8 mg/kg had low-level mutated *HER2* at baseline, which increased over time (from 1.0% to 4.2%), suggesting the development of a clone harboring mutated *HER2*.

**TABLE 1.**
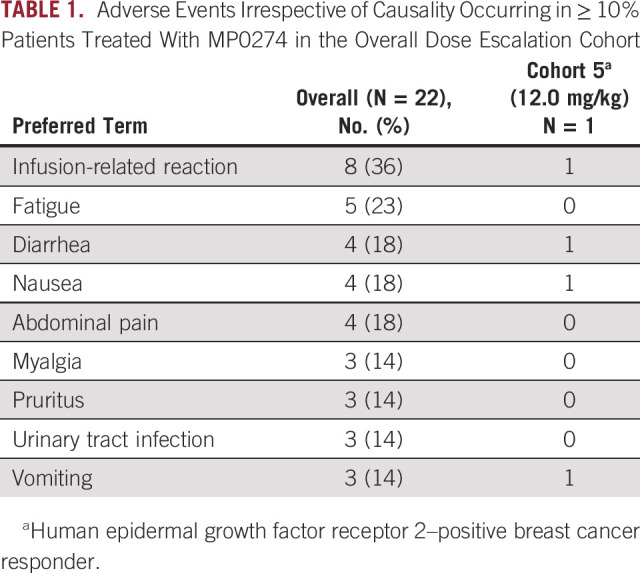
Adverse Events Irrespective of Causality Occurring in ≥ 10% Patients Treated With MP0274 in the Overall Dose Escalation Cohort

**TABLE 2.**
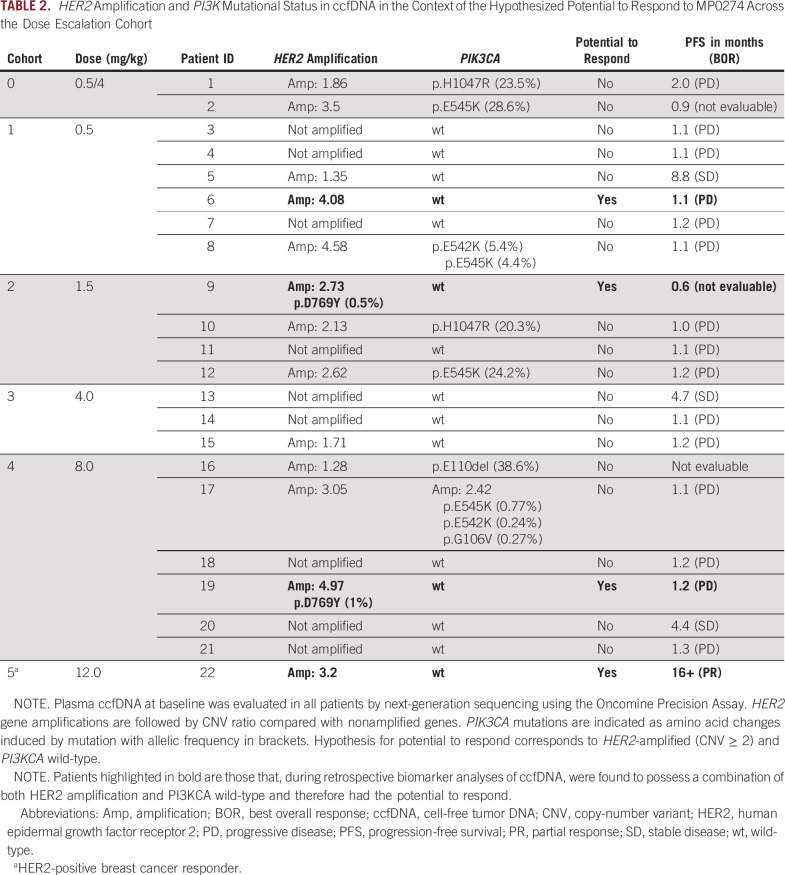
*HER2* Amplification and *PI3K* Mutational Status in ccfDNA in the Context of the Hypothesized Potential to Respond to MP0274 Across the Dose Escalation Cohort

**FIG 3.**
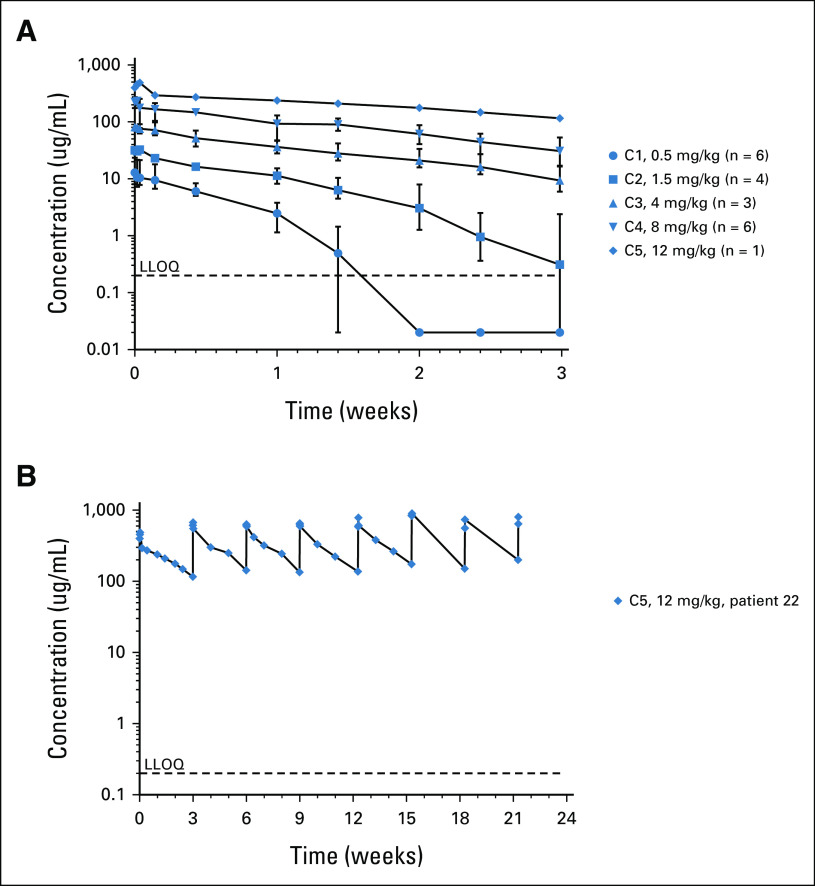
MP0274 pharmacokinetics profile. (A) MP0274 serum concentration-time profiles in the dose escalation cohorts showing geometric mean, maximum, and minimum concentrations, following the first infusion in patients treated in cohorts 1 to 5 at dose levels of 0.5, 1.5, 4.0, 8.0, and 12.0 mg/kg (n = 20). LLOQ represents the lower limit of quantification of the MP0274 bioanalytical assay (LLOQ = 0.2 ug/mL). Samples with concentrations below LLOQ were assigned a value of 0.02 ug/mL (10× lower than LLOQ). (B) MP0274 exposure in the HER2+ breast cancer patient over the first eight infusions of MP0274 at 12 mg/kg once every 3 weeks. HER2, human epidermal growth factor receptor 2; LLOQ, lower limit of quantification.

PK analyses of the first cycle across the dose escalation population showed non–dose-proportional PK for MP0274, with exposure increasing more than proportionally with increased dose and increasing drug half-life (Fig 3A). Among the six patients treated at 8 mg/kg MP0274, trough concentrations measured before dose in the second cycle ranged from 16.5 to 53.2 μg/mL. For our patient with breast cancer, the only patient treated at 12 mg/kg, the trough concentration was 116 μg/mL, with a long half-life of 15.3 days measured during the monoexponential elimination phase from 24 hours onward, and slight accumulation following repeated infusions at 3-week intervals (Fig 3B). This patient is the only patient to report a tumor response with MP0274.

## Discussion

We report here, to our knowledge, the first demonstration of clinical proof of concept for MP0274, with a sustained clinical response in a young patient with metastatic breast cancer treated at a dose level that achieved the high and sustained MP0274 concentrations predicted for required potential efficacy, after failure on two prior lines of therapy, both including an anti-HER2 monoclonal antibody (however, an anti-HER2 tyrosine kinase inhibitor was not administered). Three patients enrolled in the dose escalation study met the hypothesized optimal genetic criteria of *HER2*-positivity and wild-type *PI3KCA*; however, they were treated at dose levels likely insufficient for effective MP0274 exposure. The response to MP0274 has proven durable with ongoing tumor shrinkage reaching 78%, and our patient continued to derive benefit for 17 months after initiating therapy with MP0274. The favorable tolerability of MP0274 seen in our patient reflects the generally well-tolerated safety profile across all patients, with the main toxicity being manageable infusion-related reactions and minimal treatment delays or reductions. The response seen in this patient is consistent with the hypothesis developed on the basis of preclinical data, as well as biomarker and PK data emerging during dose escalation, that a genetic profile displaying *HER2* amplification or overexpression along with wild-type *PI3K* are both favorable and likely necessary to achieve efficacy with MP0274. With the PK analysis supporting that 12 mg/kg was potentially the lowest relevant dose for efficacy at the trough concentrations, this responding patient was the only treated patient to have both the likely optimal genetic profile and an effective MP0274 dose (peak and trough concentrations were estimated from literature reports^11-13^ and preclinical in vivo data^10^).

Our responder is, to our knowledge, the first patient treated with MP0274 to have exceeded all such potentially relevant trough concentration levels, supporting preclinical rationale for an efficacious clinical dose. Preclinical data suggest that MP0274 induces apoptosis by stabilizing the AKT-regulated fork-head transcription factor FOX03a (unpublished data), a mechanism of action that might be unique to MP0274. Although trastuzumab deruxtecan was recently approved by the US Food and Drug Administration and European Medicines Agency in the third line for metastatic *HER2*-positive patients, with convincing results emerging in the second-line setting,^14^ the mechanism of action and good tolerance seen with MP0274 may offer new and additional treatment options for patients whose tumors harbor an appropriate genetic signature. In summary, the exceptional prolonged response in our heavily pretreated patient described here is consistent with our hypothesis that HER2-positivity and *PI3K* wild-type at baseline constitute the proposed genetic signature for antitumor activity, coherent with the suggested mode of action of MP0274. PK simulations of the escalation cohorts further support that 12 mg/kg MP0274 administered once every 3 weeks reaches the required target exposure to achieve an effective biological outcome. Our experience highlights the importance and potential added value of routine sequencing in patients with HER2-positive breast cancer during the course of treatment with anti-HER2 agents to help define the best maintenance of treatment effect, as this may serve as a potential surrogate marker for treatment response and relapse.

## Figures and Tables

**TABLE A1.**
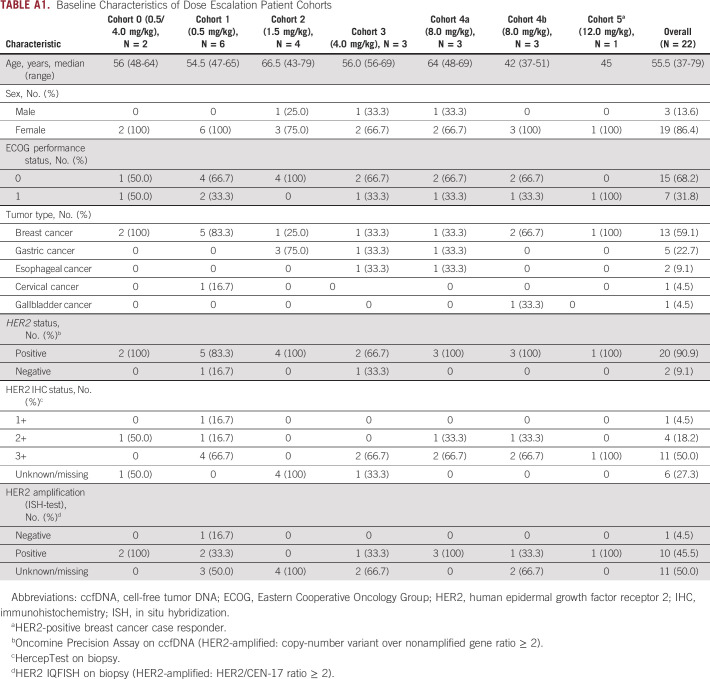
Baseline Characteristics of Dose Escalation Patient Cohorts
